# The Impact of Overstocking and Negative Energy Balance on Quantitative Measurement of Non-typhoidal *Salmonella* in Periparturient Dairy Cattle

**DOI:** 10.3389/fvets.2022.779900

**Published:** 2022-02-17

**Authors:** Lohendy Muñoz-Vargas, Jessica A. Pempek, Kathryn Proudfoot, Maurice L. Eastridge, Päivi J. Rajala-Schultz, Thomas Wittum, Gregory Habing

**Affiliations:** ^1^Department of Veterinary Preventive Medicine, College of Veterinary Medicine, The Ohio State University, Columbus, OH, United States; ^2^Department of Animal Sciences, College of Food, Agricultural and Environmental Sciences, The Ohio State University, Columbus, OH, United States

**Keywords:** *Salmonella*, overstocking, non-esterified fatty acids, cattle, periparturient

## Abstract

Stressful conditions in animal production facilities may exacerbate the fecal shedding and foodborne transmission of non-typhoidal *Salmonella enterica subsp. enterica*. Dairy cows are reservoirs of this zoonotic microorganism, and its prevalence has roughly doubled in the past decade on dairy farms in United States. Dairy cows are commonly overstocked at the feed bunk, and stressors placed on the animal prior to parturition may exacerbate *Salmonella* shedding. However, no studies have evaluated the impact of overstocking and metabolic stress on fecal concentrations of the pathogen. Therefore, we conducted a field trial with 120 multiparous dairy cows randomized into one of four treatment groups with different stocking densities at the feed bunk during the periparturient period as follows: **US**, understocked from −60 to −1 DRC; **OS**, overstocked from −60 to −1 DRC; **USOS**, understocked from −60 to −26 DRC/overstocked from −25 to −1 DRC; and **OSUS**, overstocked from −60 to −26 DRC/ understocked from −25 to −1 DRC. Fecal and blood samples were collected at four time points relative to calving. qPCR assays were used to quantify *Salmonella invA* gene and total bacterial community from fecal samples, and a subset of isolates recovered from fecal bacterial culture were characterized using pulsed field gel electrophoresis and serotyping. Serum non-esterified fatty acids (NEFA) were measured as a metabolic stress indicator using an immunoassay. Multivariable analyses were performed to test if changes in *Salmonella* concentrations were associated with stocking density, energy balance, or days relative to calving. From fecal isolates, three *Salmonella* serovars were identified, *S*. Cerro; Kentucky; Meleagridis. Concentrations of *Salmonella* increased as cows approached calving. Higher stocking densities at the feed bunk did not impact total bacterial community or NEFA; however, cows in the overstocked groups had higher *Salmonella* fecal concentrations. Further, cows with higher NEFA concentrations after calving had a higher likelihood of detection of *Salmonella*. Future farm interventions should aim to reduce environmental and metabolic stress during the periparturient period to decrease the dissemination of *Salmonella* to cattle, the environment, and humans.

## Introduction

Non-typhoidal *Salmonella enterica subsp. enterica* (from here referred as *Salmonella*) is the most frequent cause of foodborne related-deaths and hospitalizations in the United States, and in the last few decades, most human outbreaks have been associated with antimicrobial resistant strains (1–3). Multi-drug resistant strains of *Salmonella* are frequently transmitted between food producing animals and humans (4–7), including strains resistant to critically important antimicrobials such as 3rd generation cephalosporins and fluoroquinolones (8–10).

Dairy cattle are reservoirs of *Salmonella*, and although there have been strategies to decrease the prevalence of foodborne pathogens on-farm, the prevalence of *Salmonella* has roughly doubled in the past decades on dairy farms in United States (11, 12). This presents a threat to food safety, since cull dairy cows contribute significantly to the U.S. beef supply, and environmental dissemination of *Salmonella* can result in contamination of other foods. This increase in prevalence may be attributed to pathogen-associated factors, such as the emergence of cattle-adapted strains (e.g., *S*. Cerro) (13–15), or animal factors, such as chronic stress and immunosuppression. For instance, overstocking is a common management practice during the pre-partum period (12, 16, 17) and has been associated with reduced feeding time, increased rate of feed intake (18), increased competition (19), and increased plasma non-esterified fatty acid (NEFA), plasma glucose, and fecal cortisol metabolite concentrations (20). Understanding the epidemiology of *Salmonella* in dairy cows is necessary to design interventions to decrease shedding at the farm level to protect human and animal health.

The periparturient period is defined as the period from 3 weeks pre-partum to 3 weeks-postpartum and is a stage of abrupt changes on the bovine host-defense mechanisms. Prior epidemiological research has shown that the number of dairy cows shedding *Salmonella* increases around calving (21, 22) which can lead to food and environmental contamination, and human infections. However, to our knowledge, no previous studies have quantified the temporal changes in fecal concentrations of *Salmonella* in periparturient dairy cows, or determined if metabolic stress or increased stocking density exacerbate increases in fecal concentrations of *Salmonella* during the periparturient period. Therefore, the objectives of this randomized field trial were to: (1) quantify changes in fecal concentrations of *Salmonella* through the periparturient period, and (2) measure the impact of stocking density and energy balance on the changes in fecal concentrations of *Salmonella*.

## Materials and Methods

### Study Design

**Figure 1:**
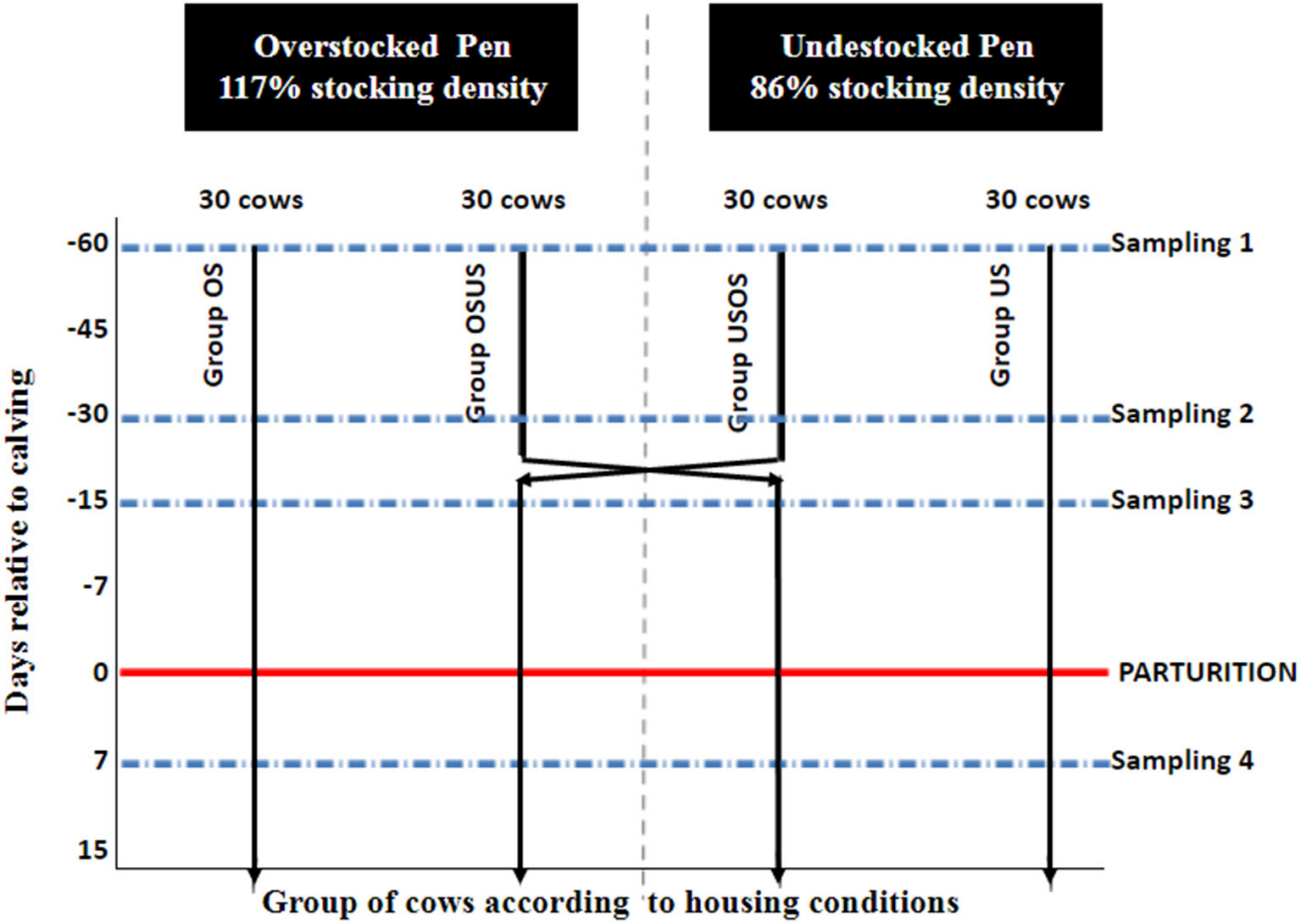
Schematic representation of pen layout according to days relative to parturition (−60, −30, −15, −1). OS, overstocked; OSUS, overstocked/understocked; USOS, understocked/ovestocked; US, understocked represent treatment groups. USOS and USOS groups were switched into adjacent pens at 26 days previous parturition.

This randomized field trial included 120 multiparous Holstein cows from a single commercial dairy farm in Northeast Ohio. Approval to conduct the study was obtained from The Ohio State University Institutional Animal Care and Use Committee (Protocol No. 2014A00000063). All enrolled cows ceased lactation (i.e., “were dried-off”) ~60 d (mean ± SD: 60 ± 8 d) prior to their expected calving date, which was calculated based on artificial insemination records. Cows were randomized to one of four treatment groups and balanced by expected calving date, parity (3.0 ± 1.1), previous 305-d mature-equivalent milk yield (39.4 ± 7.3 kg), and sire identification, respectively, in priority order (Table 1). The four groups (*n* = 30 cows/group) were housed in two adjacent pens with two treatment groups per pen. Treatment and pen design are depicted in Figure 1. Stocking densities were classified as **Overstocked** (0.86 headlocks/cow, 117% density) and **Understocked** (1.17 headlocks/cow, 86% density); the stocking densities at the feed bunk were based on common stocking densities used by commercial dairy producers (12, 17). Thirty cows of each pen were crossed over to the adjacent pen at −26 days relative to calving (DRC); therefore, the pre-calving period was divided into the “far off” (−60 to −26 DRC) and “close up” (−25 to calving) periods. Experimental groups were defined as follows: **US**, understocked from −60 to −1 DRC; **OS**, overstocked from −60 to −1 DRC; **USOS**, understocked from −60 to −26 DRC/overstocked from −25 to −1 DRC; and **OSUS**, overstocked from −60 to −26 DRC/understocked from −25 to −1 DRC. All cows had free access to feed and water, and the ratio of deep-bedded stalls to cows was approximately equal to 1:1 (range = 1.03:1–1.35:1). The same pieces of farm equipment were designated to distribute feed or collect manure throughout the study, and therefore, environmental exposure to *Salmonella* and other commensal bacteria was expected to be similar. Cattle in all groups underwent the same diet change at −26 DRC. Diet composition during the far-off period included mainly grass haylage, grass hay, and corn silage, and the diet during the close-up period included grass hay, ground wheat straw, corn silage, brewers grain, soybean meal, and protein supplements. Cows were moved to a group maternity pen as soon as signs of impending parturition were evident. After parturition, all cows were fed the same diet, with increased energy and decreased fiber content.

**Table 1:** Covariates by treatment group according to parity and 305-d milk in 120 cows.

Treatment group^*^	***N***	**Parity**	**SD**	**Milk weight (Kg)**	**SD**
OS	30	3.2	1.1	39.0	6.9
US	30	3.0	1.0	39.0	8.0
OSUS	30	2.9	0.9	39.0	7.4
USOS	30	3.1	1.2	40.5	6.9
*Mean*		3.0	1.0	39.4	7.3

* *US, understocked from −60 to −1 DRC; OS, overstocked from −60 to −1 DRC; USOS, understocked from −60 to −26 DRC/overstocked from −25 to −1 DRC; and OSUS, overstocked from −60 to −26 DRC/ understocked from −25 to −1 DRC*.

### Sample Collection

Fecal and blood samples were collected after cows were randomized and assigned to the groups at the beginning of the study (−60 DRC, range = −57 to −63), before (-30 DRC; range = −26 to −34) and after (−15 DRC; range = −13 to −19) the cross-over, and 7 days post-partum. Approximately 10 gr of feces per cow were collected via rectal retrieval using a plastic sleeve and placed individually in a sterile 18-oz Whirl-Pak^®^ bag (Nasco, Fort Atkinson, WI), transported to the laboratory on ice, and stored at −80°C. Fecal samples were used for extraction of total genomic DNA that was used as template in two real time PCR (qPCR) assays to measure the concentrations of *inv*A (*Salmonella* invasion gene) and *16s* rRNA (for total fecal bacterial quantification); the qPCR assays were adapted from previous studies (23–25). Blood samples were obtained via coccygeal venipuncture using a vacutainer system in a 10 ml blood clot collection tube (BD, Franklin Lakes, NJ), placed immediately on ice, and transported to the laboratory. Serum was obtained by centrifugation at 20.817xG for 15 min, transferred to 2 ml microcentrifuge tubes, and stored at −20**°**C until used for measurement of NEFA as indicator of metabolic stress and energy balance. NEFA from blood serum were measured for each cow at −60, −30, −15, and +7 DRC, and were quantified following a Wako Diagnostics protocol (Wako Pure Chemical Industries, Osaka, Japan) using a Spectramax M2^®^ spectrophotometer (Molecular Devices, CA, USA).

### *Salmonella* Isolation From Fecal Samples

To characterize *Salmonella* strains on the farm, a culture protocol for isolation was performed for all fecal samples collected at −60, −15, and +7 DRC. Briefly, 4 g of feces were enriched into 36 mL Tetrathionate Broth (TTB) (BD Co., Spark, MD). After incubating at 37°C for 18–24 h, 0.1 mL TTB were pipetted into 10 mL Rappaport-Vassidialis (RV) broth (BD Co.), and incubated at 42°C for 18–24 h. The following day, RV was streaked into Xylose-Lysine-Tergitol-4 agar plates (Remel, Lenexa, KS), followed by an overnight incubation at 37°C. From each positive fecal sample, a single *Salmonella* colony was transferred into MacConkey agar (BD Co., Spark, MD), and confirmed by inoculation of triple sugar iron slants, and urea broth. A slide agglutination test using polyvalent and specific antisera (Cedarlane, Burlington, NC, USA) was used to identify the serogroup of recovered isolates.

### Molecular Characterization of *Salmonella* Isolates

#### Pulsed-Field Gel Electrophoresis and Serotyping

To characterize the population of *Salmonella* on this dairy farm, a subset of 36 *Salmonella* isolates recovered from feces were selected and subjected to pulsed field gel electrophoresis (PFGE). Selected isolates represented cows from all treatment groups (*n* = 9 per group), and included samples from −60, −15, and +7 DRC (*n* = 12 per time point) that had been identified with a different serogroup based on the slide agglutination test. PFGE was performed according to Hunter et al. (26). Briefly, agarose plugs with intact DNA from *Salmonella* isolates and *S*. Branderup strain H9812 (molecular size marker) were digested using the Xba1 restriction enzyme (Roche Diagnostics, Mannheim, Germany). Electrophoresis was performed in a CHEF-DRIII chamber (Bio-Rad Laboratories, Hercules, CA) for 20 h. Similarities of banding patterns were evaluated using GelCompar II 6.6^®^ software (Applied Maths, Ghent, Belgium). Dendrograms were created using the Dice coefficient of similarity and the unweighted pair group method with arithmetic mean (UPGMA) with clustering settings of 1.5% optimization and 1.5% band position tolerance. A total of six *Salmonella* isolates, two from each distinguishable PFGE banding pattern were submitted to the National Veterinary Services Laboratory in Ames, IA for serotyping. For the purposes of this study, isolates with an indistinguishable pulsotype within equal serogroup were presumed to be the same serotype.

### Quantitative Measurements by Real Time PCR

Total genomic DNA was extracted from 0.2 g of individually homogenized fecal samples using the QIAamp Fast DNA Stool Mini Kit (Qiagen, Hilden, Germany) following the manufacturer‘s instructions, using a final reconstitution in 150 μl of elution buffer. Extracted DNA was stored at −80°C until qPCR analysis. Control DNA templates and standard curves for each qPCR assay were generated. ***For quantification of invA*, ***Salmonella* reference strain ATCC13076 was inoculated in a MacConkey agar plate (BD Co., Spark, MD), incubated at 37°C for 24 h, and suspended in triptic soy broth (TSB) (BD Co., Spark, MD) to reach a 1 × 10^8^ cell density (CFU/ml), which was measured in a Spectronic™200 spectrophotometer (ThermoScientific, Delaware, USA). Serial 10-fold dilutions were used to perform the most probable number technique (MPN) for bacterial enumeration, for which 100 μl of each dilution was plated in Xylose Lactose Tergitol 4 (XLT-4) agar plates (Remel, Lenexa, KS) in triplicate followed by an overnight incubation at 37°C. Enumeration of colonies in XLT-4 plates was obtained based on the U.S. Food and Drug Administration guideline (27). In addition to the MPN technique, 200 μl of 1 × 10^8^ dilution was used to spike 0.2 g of a *Salmonella* culture negative fecal sample previously autoclaved at 121°C/15 min. Total fecal DNA of spiked sample was extracted with a QIAamp fast DNA stool mini kit (Qiagen, Holden, Germany), and DNA quality and concentration was measured in a fluorospectophotometer (NanoDrop3300, ThermoScientific, Delaware, USA) and in fluorometer (Qubit, ThermoFisher, Delaware, USA), respectively. Then, 10-fold dilutions of DNA were used to generate a qPCR standard curve in a LightCycler^®^480 (Roche, Mannheim, Germany). The qPCR reactions were run in triplicate using 96 well-plates. Each 20 μl reaction contained 10 μl Quantitec Probe PCR master mix (Qiagen, Holden, Germany), 0.5 μM of each primer (Table 2), 0.2 μM of hydrolysis probe, and 1 μl of DNA template. Thermocycling conditions included 95°C/15 min, followed by 45 cycles of 94°C/15 s, 60°C/1 min (acquisition), and a cooling step of 40°C/10 s. Negative and positive controls were included with all qPCR assays and Cq values were evaluated to control for plate variability. Samples that generated a Cq value were classified as *Salmonella* positive. Quantification of *invA* copies per gram of feces was obtained using the slope (−3.3) and intercept generated by the standard curve with a calculated efficiency of 98%. A similar protocol was used for ***quantification of 16s rRNA*** using extracted DNA from a pure *Salmonella* isolate that was considered as positive control for the conventional PCR that targeted a region of 188 bp (Table 2). To avoid fungi quantification, primers and probe were queried against the GeneBank database. Amplicon was generated using 65°C/1 min as the annealing condition, and was used later as a template for the qPCR standard curve, generating a slope of −3.3 in a 99% efficient assay. Amplifications were performed with 95**°**C/15 min, 40 cycles of 94**°**C/15 s, 65**°**C/1 min (acquisition), and 40**°**C/10 s. Average triplicate Cq values and standard curves were used to estimate the gene copy number per gram of feces per sample for the two assays.

**Table 2:** Primer sequences used for quantification of *16s rRNA* and *invA* genes in fecal samples from dairy cows.

**Gene**	**Primer**	**Primer sequence**	**Amplicon size**
16*srRNA*^a^	Forward	5′-TACCTGGTCTTGACATCCACGGAA-3′	188 bp
	Reverse	5′- TATCACTGGCAGTCTCCTTTGAGT-3′	
	Probe	5′-/56-FAM/ATGTGCCTT/ZEN/CGGGAACCGTGAGACAGGT/3IABkFQ/-3′	
*invA* ^b^	Forward	5′-AAACGTTGAAAAACTGAGGA-3′	128 bp
	Reverse	5′-TCGTCATTCCATTACCTACC-3′	
	Probe	5′-/56-FAM/TCTGGTTGA/ZEN/TTTCCTGATCGCA/3IABkFQ/-3′	

a *GenBank accession number EU014689*.

b *NCBI Reference Sequence: NP_461817.1. Primers described previously by Hoorfar et al. (24). All probes and primers were designed using Integrated DNA Technologies, Coralville, IA*.

### Statistical Analysis

All statistical procedures were performed using SAS^®^ version 9.4 (SAS Institute, Inc., Cary, NC). Fecal concentrations of *Salmonella invA* gene were standardized to *16s rRNA* to describe the observed changes in copy numbers relative to the copy number for the entire bacterial community. Standardized concentrations were obtained by subtracting the log_10_ *16s rRNA* from the log_10_ *Salmonella* estimates (23). To perform quantitative descriptive and analytic procedures on the standardized concentrations with inclusion of cattle that were negative for *Salmonella*, an assumed value of 1 *invA* copy/g was used as the numerator for those sample concentrations. Linear mixed regression models were constructed to evaluate the effect of treatment group and time relative to calving for quantitative measures of *Salmonella*. The primary response variables were the standardized and non-standardized log-transformed values for *Salmonella*. Fixed effects considered for inclusion in the model were treatment group (OS, US, OSUS, and USOS), DRC (−60, −30, −15, and +7), and their interaction. To account for repeated measurements across time and the statistical dependence of cows within groups, DRC was included in a “repeated” statement, and cow within group was included as a random intercept. A first order autoregressive structure was selected based on the lowest Bayesian information criterion. Additionally, the 1*6s rRNA*, NEFA concentrations were log_10_ transformed and used as response variables of interest in a model with identical structure. For all models, the normality of the residuals was visually assessed. *P* ≤ 0.05 were used to declare statistical significance for all analyses. Differences in *Salmonella* concentrations between treatment groups at single time points, and the treatment group association with the *Salmonella* concentration differences between time points were compared using a Mann-Whitney test. To identify the association between *Salmonella* culture results (negative or positive) and NEFA concentrations (high or normal) a logistic regression model and a pairwise comparisons of LSMeans using a generalized linear mixed model were performed. NEFA values were dichotomized as high or normal based on previously established thresholds, in which 0.26 mEq/L was used as threshold for −60 and −30 DRC, 0.29 mEq/L for −15 DRC, and 0.57 mEq/L at +7 DRC (28–30). The model was constructed with a random intercept with cow nested within treatment group accounting for repeated measures. DRC, treatment group, and the treatment group by DRC interaction were included as fixed effects.

## Results

Of the 120 cows initially enrolled in this study, 11 cows were excluded from all analyses due to subsequent diagnoses of heart failure (*n* = 1), lameness (*n* = 2), or early calving (>1 week, *n* = 8), and were not affected by parity number or prior milk production Thus, a total of 26, 28, 26, and 29 cows within the OS, US, OS-US, and US-OS, respectively, were included in the analysis; measurements were available for 436 samples obtained from 109 cows at −60, −30, −15, and +7 DRC.

### Characteristics of Recovered *Salmonella* Isolates

**Figure 2:**
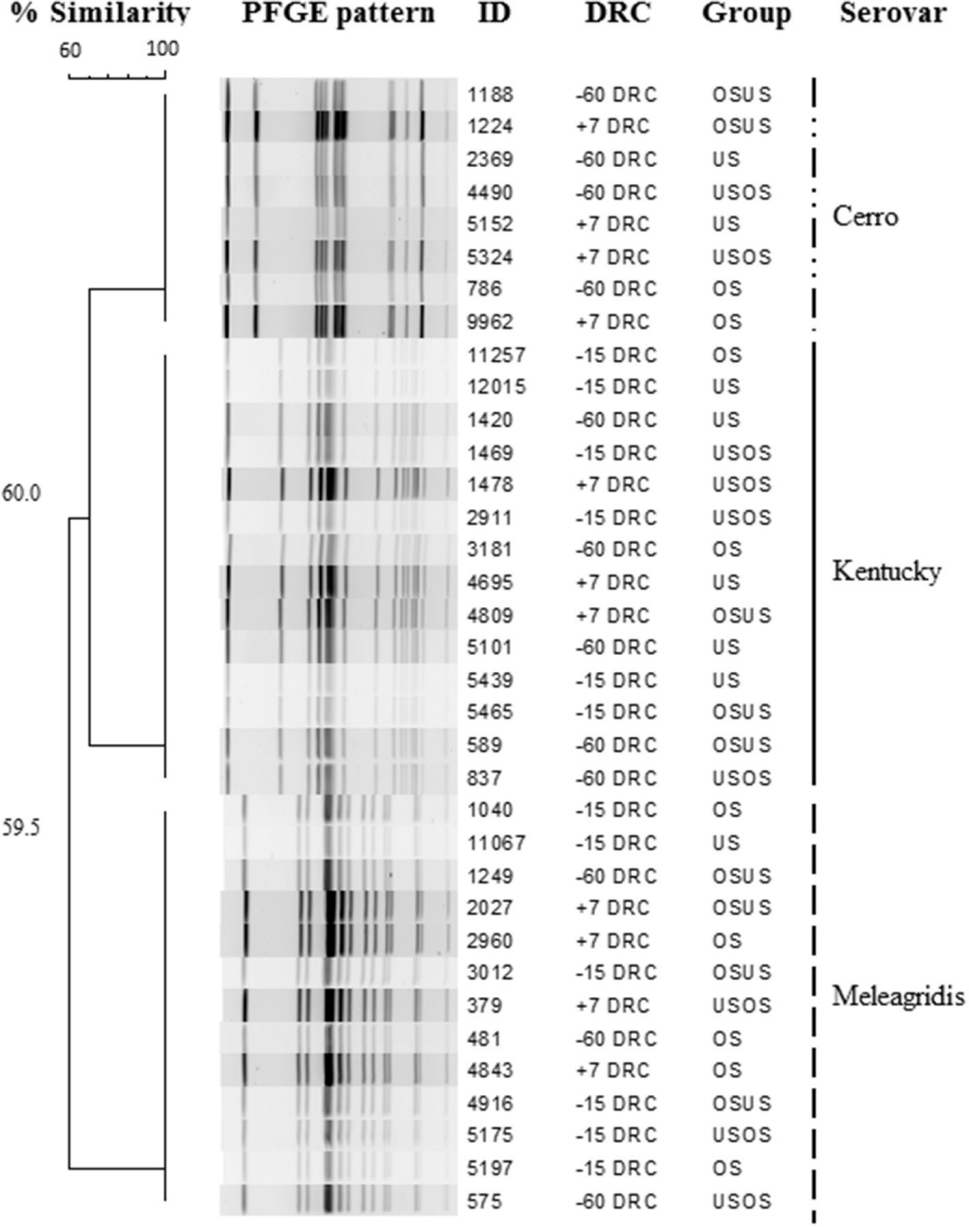
Dendrogram showing pulsed field electrophoresis profiles of *Salmonella* isolates recovered from periparturient cows according to days relative to calving (DRC) and treatment group. Three serotypes were identified among isolates.

The overall proportion of samples positive on *Salmonella* culture was 72% (236/327). A numerically higher prevalence was observed during the close-up period (−15 DRC) with 79.8% (87/109) positive samples compared to the far-off (68%, 75/109) or post-calving periods (66%, 73/109). Of the 36 isolates selected for characterization, three different PFGE patterns and three different *Salmonella* serotypes were identified, *S*. Cerro, Kentucky, and Meleagridis. All isolates with the same serotype had indistinguishable banding patterns (Figure 2) and were identified in representative samples from all time points and treatment groups, suggesting that clonal strains were equally distributed among cows and treatment groups. Additionally, the *Salmonella* concentrations did not differ (*p* = 0.90) by serovar, with 1.2 × 10^4^, 4.6 × 10^3^, and 3.5 × 10^3^ copies/g feces for *S*. Cerro, Kentucky, and Meleagridis, respectively.

### Impact of Proximity to Parturition on Fecal Concentration of *Salmonella*

**Figure 3:**
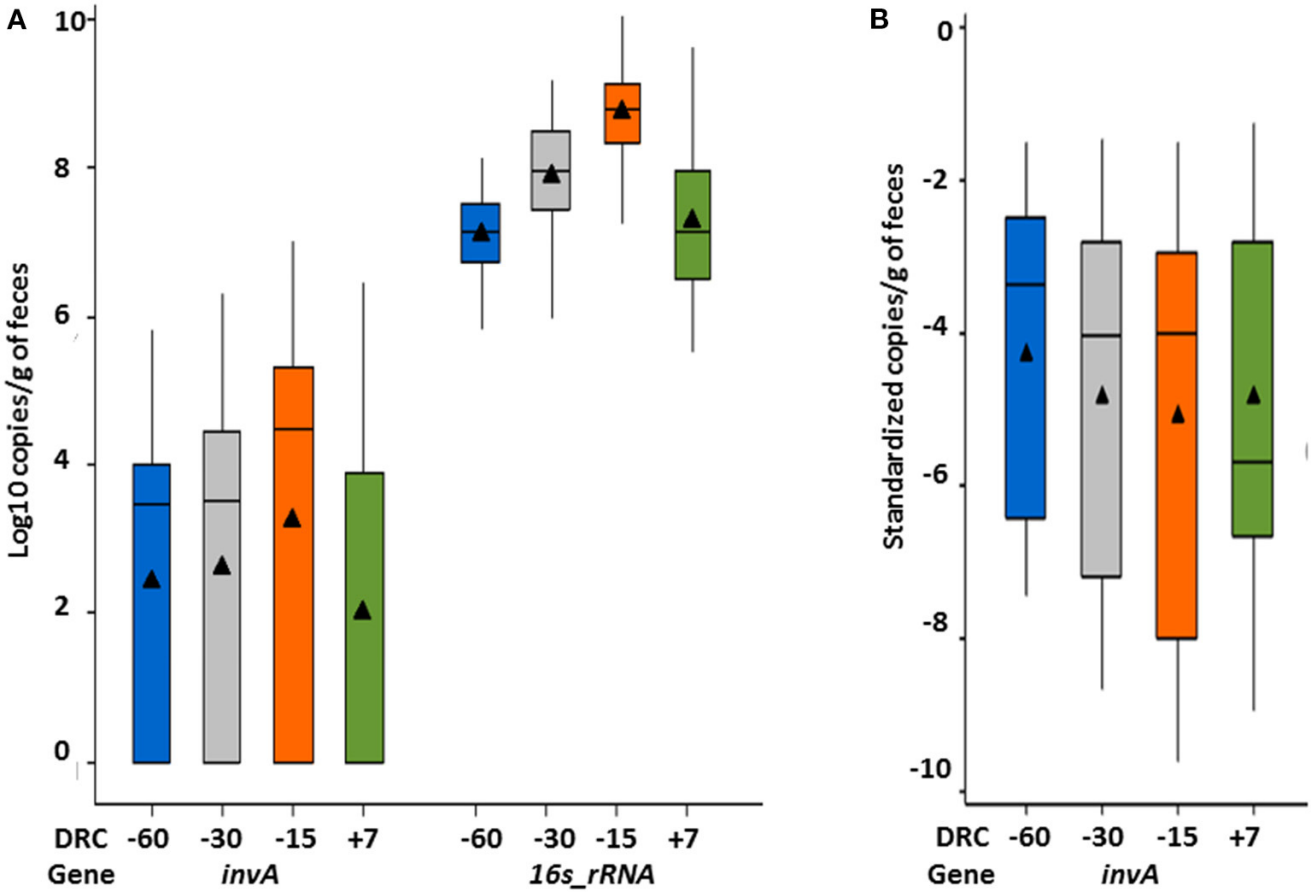
Whisker box plot representing temporal variations of **(A)** *Salmonella* (*inv*A gene) and *16s rRNA* fecal concentrations; and **(B)** *inv*A standardized concentrations relative to total fecal bacterial quantification (*16s_ rRNA* gene) during the periparturient period. Mean (solid triangle), median (horizontal line), quartiles, and highest/lowest observations are represented in each figure.

In total, 57.8% (252/436) of samples were *Salmonella* positive based on the qPCR assay that provided a minimum quantifiable limit of 75 copies/g feces. The overall mean concentration of the *invA* gene (copies/g of feces) was 1.5 × 10^5^ (median = 3.2 × 10^3^). Among the positive samples, *Salmonella* fecal concentrations ranged between 7.5 × 10^2^-1.01 × 10^7^ copies/g feces. As cows approached parturition (−15 DRC), *Salmonella* concentrations increased (Figure 3A), and was significantly different from all other time points (*p* < 0.05), with 1.76 × 10^4^ (median = 3 × 10^3^) at −60 DRC, 8.2 × 10^4^ (median = 3.2 × 10^3^) at −30 DRC, 4.3 × 10^5^ (median = 3.1 × 10^4^) at −15 DRC, and 7.3 × 10^4^ at +7 DRC. Similarly to *Salmonella* culture results, the proportion of *Salmonella* positive samples on qPCR was higher prior to parturition, and numerically lower following parturition. Across time points, 60.5, 59.6, 63.3, and 47.7% cows were positive at −60, −30, −15, and +7 DRC, respectively. Among positive cows, the median fecal concentration was 8 × 10^3^, 1.7 × 10^4^, 1.4 × 10^5^, and 9 × 10^3^ *inv*A copies/g of feces at −60, −30, −15, and +7, respectively. The median standardized concentrations of *Salmonella* were significantly different across time points during the transition period; showing significantly higher concentrations prior parturition (*p* < 0.001) as depicted in Figure 3B. However, the magnitude of differences between time points was smaller compared to the values for the non-standardized *Salmonella*.

### Impact of Stocking Density on Changes in *Salmonella* Fecal Concentrations

**Figure 4:**
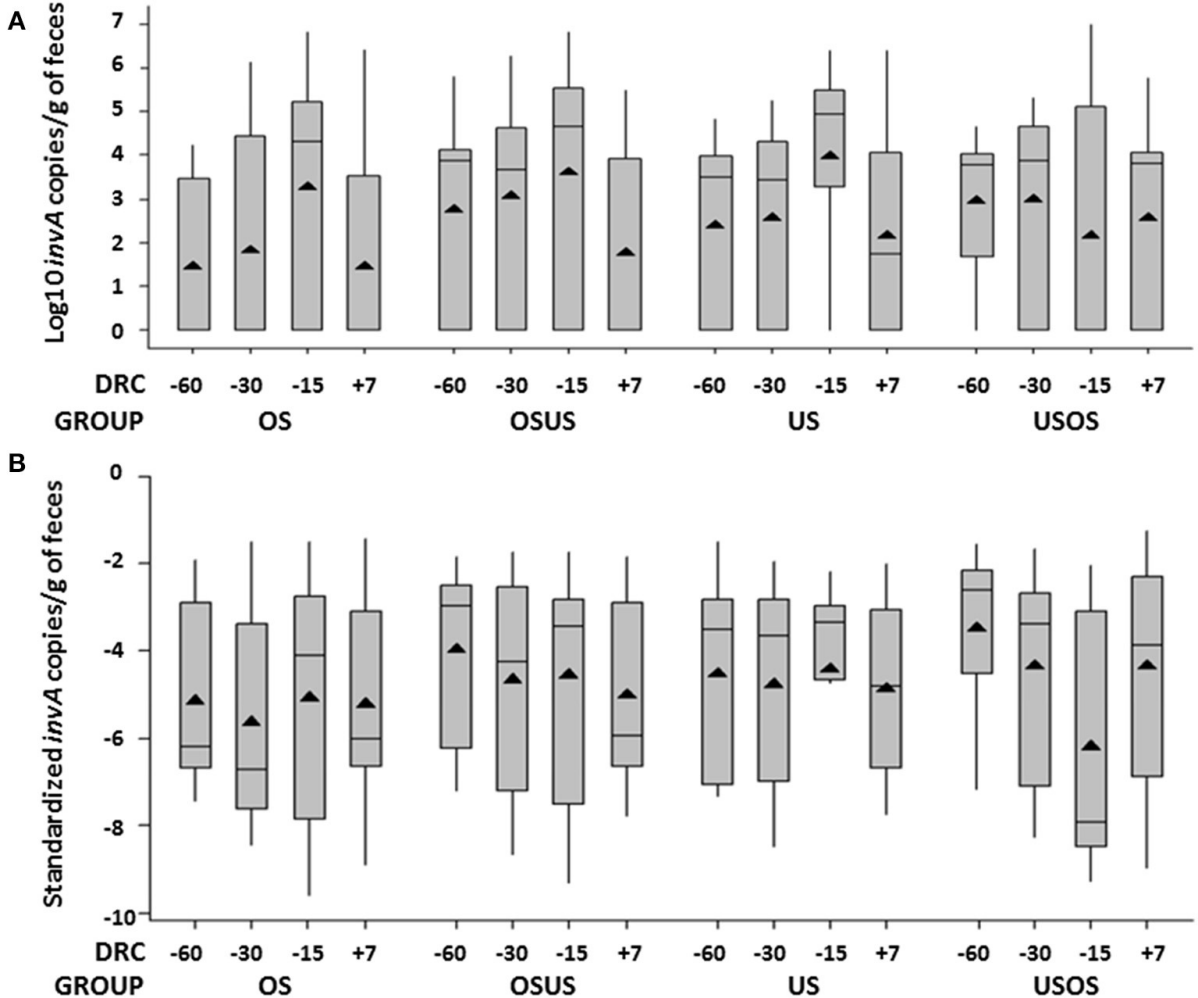
Whisker box plot representing treatment group variations per days relative to calving (DRC) in **(A)** non-standardized, and **(B)** standardized fecal concentrations of *Salmonella* (*inv*A gene). Mean (solid triangle), median (horizontal line), quartiles, and higher/lower observations are represented in each figure.

The magnitude of the change in *Salmonella* fecal concentrations through the periparturient period was larger for groups with a higher stocking density at the feed bunk (Figure 4A). Amongst treatment groups that experienced only one stocking density throughout the study period, the OS group experienced an increased mean of 5 × 10^5^ copies/g (*p* = 0.049) between enrollment (−60 DRC) and the sampling point with the highest overall *Salmonella* shedding (−15 DRC) compared to an increased mean of 2.9 × 10^5^ copies/g amongst the cows in the US group. In addition, the difference in *Salmonella* concentrations was significantly higher (*p* = 0.05) for overstocked cows between the −30 DRC and −60 DRC (8.9 × 10^4^ copies/g), than those understocked (2.1 × 10^4^). Among the treatment groups that experienced a pen switch at −26 DRC, the group that moved from a higher to lower stocking density (OSUS) experienced a decrease of 4.8 × 10^5^ mean copies/g in *Salmonella* concentrations from −15 to +7 DRC (*p* = 0.001). In contrast, the treatment group that moved to a higher stocking density (USOS) had an increase of 3.8 × 10^5^ mean copies/g between both time points. These results suggest an effect of overstocking on *Salmonella* concentrations. In addition, temporal changes were observed in overall standardized concentrations of *Salmonella* among treatment groups (*p* = 0.0039). In particular, those groups that were switched at −26 DRC approaching parturition presented differences in *Salmonella* standardized concentrations (*p* = 0.007), with mean copies/g of feces of 2.7 × 10^5^ in OSUS and 6.3 × 10^7^ in USOS groups from −15 DRC to a week after parturition (Figure 4B).

### Association Between NEFA Concentrations and *Salmonella*

**Figure 5:**
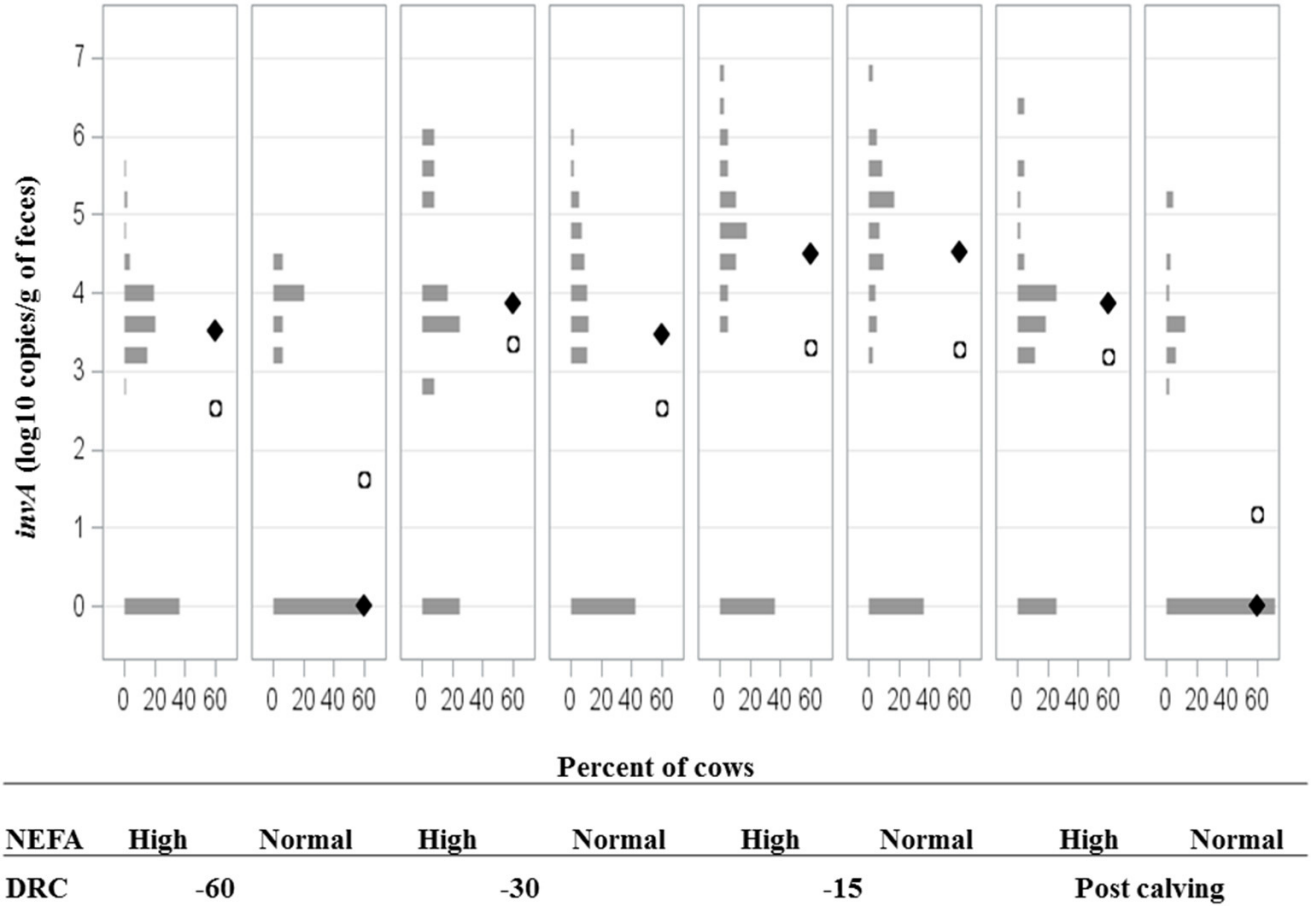
Frequency distribution of *Salmonella invA* gene fecal concentrations for dairy cattle with high or normal serum concentrations of non-esterified fatty acids (NEFA) at −60, −30, −15 days relative to calving (DRC), and post-calving. Solid diamonds and open circles represent the median and mean, respectively.

Higher NEFA concentrations (*p* < 0.001) were observed after calving compared to the far-off and close-up periods; with mean concentrations (95% CI) of 0.56 mEq/L (0.5–0.6 mEq/L), 0.17 mEq/L (0.11–0.21 mEq/L), and 0.31 mEq/L (0.26–0.35 mEq/L), respectively. However, different thresholds for NEFA concentrations have been established for the periparturient period, and therefore, values were dichotomized for the purposes of analysis based on previously established time-specific thresholds. Based on this categorization, 42.9% (187/436) of serum samples were classified has having high NEFA; a total of 85.3% (93/109) cows had high NEFA levels at −60 DRC, 11% (12/109) at −30 DRC, 36% (39/109) at −15 DRC, and 40% (43/109) after calving. Cows housed within different stocking densities did not have different NEFA concentrations, or experience different changes in NEFA concentrations over time (DRC by group interaction, *p* > 0.05). However, *Salmonella* was significantly more likely to be recovered from feces of cows with higher NEFA concentrations (Figure 5). Overall, 49.6% (126/254) of *Salmonella* positive cows had a high NEFA serum concentration (0.41 mEq/L, 95% CI = 0.37–0.45), compared to 33% (61/184) of cows with a *Salmonella-* negative samples (0.32 mEq/L, 95% CI = 0.27–0.36). As shown in Table 3, significant differences in NEFA concentrations between *Salmonella* positive and negative samples were observed after calving (*p* < 0.001).

**Table 3:** Least square means (95% CI) for 436 non-esterified fatty acids (NEFA, mEq/L) measurements according to qPCR *Salmonella* status (positive or negative) during the periparturient period of 109 cows sampled at four time points.

**Days relative to calving (DRC)**	**NEFA mean (*N* = 436)**	***Salmonella* positive (*n* = 252)**	***Salmonella* negative (*n* = 184)**	*P*-value^*^
−60	0.46	0.5 (0.43–0.56)	0.4 (0.33–0.48)	0.08
−30	0.17	0.18 (0.11–0.24)	0.14 (0.07–0.22)	0.5
−15	0.31	0.3 (0.24–0.36)	0.29 (0.2–0.38)	0.7
7	0.56	0.74 (0.67–0.8)	0.39 (0.33–0.45)	<0.001

* *p-values obtained by pairwise comparisons of LSMeans using a generalized linear mixed model*.

### Quantification of Bacterial Population

Of the 436 samples, 7% contained 1 × 10^5^ copies of *16s rRNA* gene per gram of feces, followed by 30.7, 32, 26, and 3.9% with 1 × 10^6^, 1 × 10^7^, 1 × 10^8^, and 1 × 10^9^ copies. Among all cows, the overall mean concentration was 1.5 × 10^8^ copies/g. Total bacterial community did not differ across stocking densities (*p* = 0.46), and there was not a treatment by time interaction (*p* = 0.60). However, similarly to *Salmonella* concentrations, significantly higher (*p* < 0.001) fecal concentrations of *16s rRNA* were observed at −15 DRC with 2 × 10^8^ copies/g (1.6 × 10^8^-3.2 × 10^8^), compared to 2.8 × 10^7^ copies/g (2 × 10^7^-4 × 10^7^) at far-off and 7 × 10^6^ copies/g (5 × 10^6^-8 × 10^6^) post-calving (Figure 3A).

## Discussion

### *Salmonella* During the Transition Period

Considerable concern exists regarding the proliferation and dissemination of zoonotic pathogens commonly found in animal settings, and the role that common practices applied for the animal production contribute on this spread. In this study, we particularly evaluated the impact of proximity to parturition on the prevalence and fecal concentrations of *Salmonella*, and additionally aimed to measure the impact of increased stocking density at the feed bunk on the exacerbation of fecal concentrations of this microorganism. To our knowledge, this the first study to quantify the temporal changes of *Salmonella* in dairy cows. Because it was conducted on a large commercial dairy farm, aspects of our study design (e.g., the number of pens per treatment group) were limited. Nonetheless, our study reports novel cow-level data regarding fecal *Salmonella* serotypes and concentrations as cows approach calving.

Four treatment groups of cows approaching parturition were monitored from over 2 months period. Increases in fecal detection and concentrations of *Salmonella* were observed around the calving day. The trend of higher prevalence of *Salmonella* in cattle approaching parturition has been observed in previous studies, mostly detected by culture methods, and have suggested this period as a risk factor for pathogen shedding and metabolic diseases. The absolute *invA* gene data were normalized to measurements of the *16s rRNA* concentrations to determine the abundance of *Salmonella* relative to the whole bacterial community. An increase in *Salmonella* coincided with an increase in the total bacterial population as cows approach parturition. The total fecal bacterial community substantially increased (log 10) 2 wk pre-calving relative to dry-off. These results are not surprising since changes in fecal bacterial abundance and diversity in periparturient cows were described previously (31) and could be associated with production practices, including diet changes and pen movement. The impact of nutrition and management practices during this transition period have been extensively studied, mainly to determine the impact on health conditions, such as hepatic lipidosis (32), reproductive disorders (33, 34), mastitis (35, 36), and milk fever (37). Yet, there is scarce scientific literature regarding the impact of this period on infectious agents or the dissemination of microbial hazards, such as *Salmonella*. Disruptions in the gut microbiome can enhance *Salmonella* colonization, and variations in the community structures can be identified between healthy and diseased animals and before and after antimicrobial therapies or changes in diet (38–43). In a healthy microbiome, high concentrations of short chain fatty acids (SCFAs) represent a significant nutrient source that can be used to enhance host defenses. Recent studies have demonstrated that SCFAs suppress *Salmonella* infection via inflammasome activation (44). Thus, SCFAs are promising substances for dietary supplementation in animals with disrupted microbiome for which numerous metabolic and hormonal pathways can be affected, including the immune system regulation. Additionally, others (41) evaluated the impact of diet changes during the periparturient period on ruminal microbiome reporting significant increases in the ratio of *Bacteroidetes and Firmicutes*, mainly in primiparous animals. In our study, changes in diet from the far-off (−60 to −26 DRC) to close-up period (−25 to −1 DRC) could have impacted the gut microbial populations contributing to bacteria proliferation observed from −30 to −15 DRC; however, all cows in all treatment groups experience the same diet change at the same time. Therefore, although the diet changes may explain overall temporal differences, the diet changes do not explain between-group differences in the magnitude of changes in shedding through the periparturient period. Decreased concentrations of the total bacterial population after parturition, suggest that other factors (i.e., fecal water content, effect of other pathogens) not assessed in this study likely contributed to the higher measured concentrations of bacteria before parturition. A recent study (45) demonstrated that some metabolites associated to taurine, hypotaurine, and arginine metabolism are enhanced at pre-partum period, stimulating proliferation of some bacterial species such as *Lactobacillus, Streptococcus*, and *Clostridium*. In contrast, other species such as *Bacteroides, Escherichia*, and *Campylobacter* are enhanced after calving through the activity of some metabolites associated to the vitamin B6 and glycerophospholipid metabolism. This suggests that increases in bacterial concentrations observed before parturition in the present study could be favored by the diversity and community structure of the periparturient cows gut microbiome and the stimulation of some specific metabolites. For instance, taurine has been identified as an important regulator for the intestinal microbiota and SCFAs production (46), providing gut health by inhibiting pathogen growth. This is interesting and could be paradoxical since beneficial metabolites such as butyric, acetic, and lactic acid that could justify the increased microbial quantification at −15 DRC could also have an inhibitory effect on *Salmonella* proliferation (44), suggesting that others factors besides diet were also involved on the observed microbial dynamics.A variety of serotypes have been described across a diverse compendium of sample types from cattle (47), many recovered from fecal samples of seemingly healthy animals and lymphatic tissues at abattoirs (31). In the present study, three serovars commonly described in cattle were observed homogeneously distributed regardless the treatment or the period relative to calving, including *S*. Meleagridis, Cerro and Kentucky, all previously isolated from ground beef for human consumption (48) and linked to laboratory-confirmed human illnesses (1). This finding and the high *Salmonella* frequency observed across the time points represent a relevant information for farm personnel exposed to occupational activities during cattle handling and highlight the importance of personnel training and strengthening of biosecurity measures as prevention strategies against *Salmonella* infections. In addition, culled dairy cattle could represent a risk for foodborne human salmonellosis since this bacterium expresses tropism to lymphatic tissues that are difficult to surface decontaminate at slaughter and are often incorporated into ground beef as beef trim.

### Effect of Metabolic Stress on *Salmonella*

Non-esterified fatty acids are a main source of energy to cattle, and derive from the released fatty acids from adipocytes (49). The concentration of blood circulating NEFA reflects the degree of fatty acids mobilization. Therefore, the NEFA blood concentration proportionally increases as the negative energy balance increases. In the present study, moderate increases in stocking density at the feed bunk did not alter energy metabolism during the far-off or close-up periods. However, higher plasma NEFA concentrations were associated with *Salmonella* fecal shedding, supporting our initial hypothesis. In an observational repeated sampling study (50), demonstrated that cows have higher risk of becoming infected with *Salmonella* (e.g., *S*. Dublin) during calving, especially in herds with clinical outbreaks. Further, the authors suggested that stress could be an important risk factor for infection; however, the focus of the study was to evaluate the risk of becoming a carrier after infection. According to Ospina et al. (29) ≥ 0.3 mEq/L NEFA concentrations 2 wk prior to calving is associated with increased risk of disease and decreased production after calving. The authors established the NEFA threshold at the cow-level pre- and post-partum, and predicted the risk associated between high NEFA and post-partum diseases. Despite of that no analysis of the association between NEFA on *Salmonella* was carried out in that study, it supports our finding of higher NEFA concentrations on *Salmonella-*positive cows. In our research, 83% of cows depicted high NEFA concentrations at the beginning of the study, 60 days prior parturition, suggesting metabolic stress and negative energy balance during the late lactation period. In contrast, others (30) have reported higher levels of NEFA during early lactation and decreased NEFA serum concentrations during the far-off dry period. Differences in results are due to the use of different NEFA thresholds after calving, which should be higher than at pre-parturient period. The results of the present study showed changes in NEFA concentrations associated with *Salmonella* shedding in high producing dairy cows; mostly induced by negative energy balance and lactogenesis. This metabolic indicator can be considered as a tool to assess the energy balance in dairy cows at different points around parturition, and to address herd management and production disorders in order to conduct diagnosis, treatment, and prevention of metabolic disorders and infection control.

## Conclusions

The longitudinal approach used in this study allowed for detection of changes in fecal concentrations of *Salmonella* during the periparturient period. Specifically, fecal concentrations of *Salmonella* (57.8% overall prevalence) and culture detection (72% prevalence) increased as cows approached calving. Higher stocking densities at the feed bunk did not impact total bacterial community, yet, cows within the overstocked treatment groups showed larger differences of *Salmonella* fecal concentrations as they approached parturition compared to understocked groups. This study demonstrated a higher likelihood of detection of *Salmonella* in cows with high NEFA concentrations after parturition; future controlled trials should establish thresholds of NEFA for optimal control of *Salmonella* and other microbial hazards. Farm interventions during the periparturient period should be implemented to decrease the dissemination of *Salmonella*, and reduce the potential risks associated with food safety and public and animal health.

## Data Availability Statement

The original contributions presented in the study are included in the article/supplementary material, further inquiries can be directed to the corresponding author/s.

## Ethics Statement

The animal study was reviewed and approved by the Ohio State University Institutional Animal Care and Use Committee (Protocol No. 2014A00000063). Written informed consent was obtained from the owners for the participation of their animals in this study.

## Author Contributions

LM-V: sample collection, lab processing, data analysis, and manuscript writing. JP: funding acquisition, experimental design, lab processing, and manuscript editing. KP: experimental design, sample collection, and data analysis. ME: experimental design and sample collection. PR-S: data analysis. TW: data analysis and lab processing. GH: funding acquisition, experimental design, data analysis, and manuscript editing. All authors contributed to the article and approved the submitted version.

## Funding

This research was funded in part by the Public Health Preparedness of Infectious Diseases (PHPID) grant 29-10003 at the Ohio State University, Columbus, Ohio, United States, entitled: Stress, the microbiome, and food safety: Quantifying the impact of stress in farm animals on human microbial hazards and SEEDS: OARDC Research Enhancement Competitive Grants Program grant 2014104 entitled Heat and social stress may affect the welfare and productivity of dairy cows and calves.

## Conflict of Interest

The authors declare that the research was conducted in the absence of any commercial or financial relationships that could be construed as a potential conflict of interest. The reviewer HMS declared a declared a past co-authorship with one of the authors TW.

## Publisher's Note

All claims expressed in this article are solely those of the authors and do not necessarily represent those of their affiliated organizations, or those of the publisher, the editors and the reviewers. Any product that may be evaluated in this article, or claim that may be made by its manufacturer, is not guaranteed or endorsed by the publisher.
